# Exploration of African natural products as VP35 inhibitors to combat Marburg virus infection: Molecular docking, molecular dynamics, and quantum mechanical computations

**DOI:** 10.1371/journal.pone.0334160

**Published:** 2025-10-24

**Authors:** Alaa H. M. Abdelrahman, Gamal A. H. Mekhemer, Peter A. Sidhom, Mohamed A. El-Tayeb, Shahzeb Khan, Mahmoud A. A. Ibrahim

**Affiliations:** 1 Computational Chemistry Laboratory, Chemistry Department, Faculty of Science, Minia University, Minia, Egypt; 2 Department of Pharmaceutical Chemistry, Faculty of Pharmacy, Tanta University, Tanta, Egypt; 3 Department of Botany and Microbiology, College of Science, King Saud University, Riyadh, Saudi Arabia; 4 Centre for Pharmaceutical Engineering Science, Faculty of Life Science, School of Pharmacy and Medical Sciences, University of Bradford, Bradford, United Kingdom; 5 Department of Engineering, College of Engineering and Technology, University of Technology and Applied Sciences, Nizwa, Sultanate of Oman; 6 School of Health Sciences, University of KwaZulu-Natal, Westville Campus, Durban, South Africa; Government College of Engineering, Keonjhar, INDIA

## Abstract

Marburg virus (MBV) is a highly lethal filovirus responsible for hemorrhagic fever with case fatality rates of up to 88%. MBV was first recognized in 1967 during simultaneous outbreaks in Marburg and Frankfurt, Germany, and Belgrade, then part of Yugoslavia (now Serbia), following exposure to infected African green monkeys imported from Uganda. Currently, no approved treatment exists for MBV infection. The viral protein (VP35) plays a critical role in viral replication, transcription, and nucleocapsid assembly, making it a promising antiviral target. Consequently, obstructing the function of VP35 offers a potential strategy for combating MBV. Herein, the African Natural Products (ANP) database, which encompasses over 6,500 compounds, was subjected to virtual screening against VP35 employing docking computations. For inhibitors exhibiting a docking score <−8.0 kcal/mol against VP35, molecular dynamics simulations (MDS) were conducted, along with binding energy assessment utilizing the MM/GBSA approach. Upon the MM/GBSA//250 ns MDS, ANPDB6426, ANPDB5109, and ANPDB6357 demonstrated promising binding affinities toward the VP35, with Δ*G*_binding_ values of −37.9, −34.6, and −34.2 kcal/mol, respectively. The post-MD analyses demonstrated that all three ANPs remained remarkably stable within the VP35 binding pocket over the full 250 ns MDS. Furthermore, the identified ANPs unveiled favorable oral bioavailability, pharmacokinetic, and safety profiles. Density functional theory calculations further supported the chemical reactivity of the identified ANPs. Compared to galidesivir and favipiravir, reference inhibitors, the estimated MM/GBSA binding energies of the identified ANPs with VP35 were about two times lower than galidesivir and favipiravir. These results highlighted the efficacy of computational methods in recognizing putative VP35 inhibitors, providing promising avenues for additional experimental research and prospective curative advancement toward MBV.

## Introduction

Marburg virus (MBV) is an enveloped, negative-sense RNA filovirus, sharing structural and genomic features with Ebola virus [1,2]. The emergence of the MBV, categorized within the *Filoviridae* family, has raised considerable concerns regarding public health due to its capacity to cause severe hemorrhagic fevers, causing high mortality rates [3,4]. In 1967, MBV was initially recognized in Serbia, Frankfurt, Germany, and Marburg [5]. Since this discovery, at least 15 human outbreaks have occurred across Africa, most recently in Ghana in early 2022 and Tanzania in April 2023 [6]. According to Kaifa *et al*. (2023), the Tanzanian outbreak demonstrated that MBV is re-emerging as a pathogenic zoonotic threat [7]. The lethal characteristics of the MBV can be linked to its complex interactions with the biological mechanisms of its hosts [8,9]. MBV contagion treatment is mainly dependent on alleviating symptoms and complications, while there is an urgent demand for efficient therapeutic interventions [10]. Central to the virulence of the MBV is its molecular structure, especially the array of both structural and non-structural proteins that are vital for the virus’s life cycle [11]. Over the past few years, there has been an increasing focus on the discovery of highly effective inhibitors targeting essential proteins of MBV [12,13]. These inhibitors focus on various proteins that are involved in the process of viral replication, aiming to hinder viral entry, replication, or assembly. Numerous potential candidates have surfaced as a result of these endeavors, and a subset has demonstrated effectiveness in animal models. Although several antivirals—such as remdesivir, favipiravir, galidesivir, and monoclonal antibodies—have shown efficacy against filoviruses in preclinical models, their effectiveness against MBV specifically remains limited and largely unvalidated in human clinical settings [14]. Most of these agents were developed primarily for the Ebola virus and repurposed for MBV, with only partial or inconsistent protection reported in animal studies [15]. Given these limitations, there is a critical need to discover novel, potent, and orally bioavailable anti-MBV drug candidates.

The MBV genome (~19 kb) encodes seven structural proteins (NP, VP35, VP40, GP, VP30, VP24, L), with VP35 serving a dual role as both viral polymerase cofactor and potent interferon antagonist [16]. VP35 engages with double-stranded RNA, functioning as an antagonist of interferon, which in turn obstructs the initiation of essential host immune responses that target viral invaders [17]. Such attenuation of the immune response results in clinical manifestations of MBV contagions, permitting unrestricted viral proliferation [18]. Besides, VP35 participates in viral RNA synthesis as it interacts with the RNA polymerase complex. The vital functions of VP35 underscore its prospective as a promising target for MBV treatments [19]. Concentrating on VP35 as a potential druggable target shows promise; nevertheless, an in-depth comprehension of its structural and functional dynamics is required [20].

**Fig 1:**
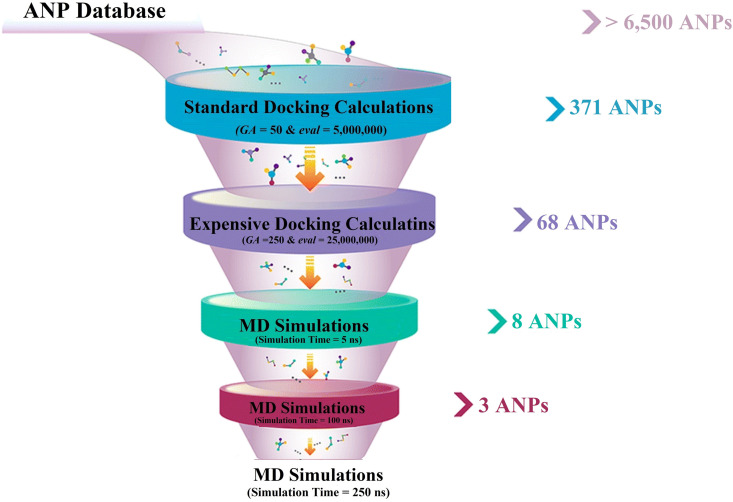
Schematic diagram of the employed computational methodologies for hunting prospective VP35 inhibitors from the ANP database.

Natural products (NPs) derived from microbial and plant origins are essential in the area of pharmaceutical advancement, particularly in combating cancer and infectious illnesses [21,22]. Numerous drugs, including paclitaxel, vinblastine, and morphine, were derived from natural sources, particularly plants [23]. Paclitaxel, isolated from *Taxus brevifolia* (Pacific yew), stabilizes microtubules and inhibits mitosis [24]. Vinblastine, derived from *Catharanthus roseus* (Madagascar periwinkle), disrupts microtubule assembly, leading to cell cycle arrest [25]. Although primarily used for paclitaxel and vinblastine in cancer therapy, their successful development underscores the pharmacological potentiality of NPs [26]. Morphine, an opioid analgesic obtained from *Papaver somniferum* (opium poppy), is widely used for severe pain management [27]. Remarkably, more than 120 NP databases have been released and are widely recognized [28]. Among these NP databases, the African Natural Products (ANP) database encompasses > 6,500 molecules primarily derived from plant sources, with additional contributions from various endophytes, fungi, and bacteria [29,30]. Herein, the ANP database was mined for hunting prospective VP35 inhibitors utilizing docking computations. Upon docking scores, the most potent ANPs underwent molecular dynamics simulations (MDS), and their respective binding energies were calculated employing the MM/GBSA approach. The medicinal chemistry and physicochemical properties of the identified ANPs were also anticipated to assess their oral bioavailability. Furthermore, density functional theory (DFT) was employed to gain a deeper understanding of the electronic and structural characteristics of the identified ANPs. The schematic diagram of the employed computational methodologies for hunting prospective VP35 inhibitors from the ANP database is illustrated in Fig 1. Collectively, these results emphasize potential candidates that may successfully target VP35 of the MBV, presenting a promising therapeutic avenue for subsequent experimental assays.

## Computational methodology

### VP35 preparation

The three-dimensional (3D) structure of VP35, with PDB code 4GH9 and a resolution of 1.65 Å, was accessed from the protein data bank and used as a query for all *in-silico* computations [31]. Prior to molecular docking, the protein target was subjected to several preprocessing procedures to confirm its appropriateness for examination. This preparation process involved the extraction of heteroatoms and water molecules, leading to an enhanced protein structure formatted in PDB. The protonation states of titratable residues were determined at a neutral pH of 7.4 using the PROPKA software [32], and the missing H-atoms were consequently added.

### ANP database preparation

The ANP database, comprising over 6,500 ANPs, was obtained from http://african-compounds.org in SDF format and prepared for virtual screening towards VP35. Utilizing Omega2 software, the 3D structures of the investigated ANPs were generated [33,34]. Energy minimization was then performed for the generated 3D structures utilizing the MMFF94S force field within the SZYBKI software to achieve the lowest energy conformation [35,36]. The replicated ANPs were excluded based on the International Chemical Identifier (InChIKey) [37]. The ionization states of the examined ANPs were inspected utilizing the fixpka application incorporated in the QUACPAC software [38]. The Gasteiger–Marsili method was utilized to determine the atomic charges of the investigated ANPs [39]. All prepared ANPs are accessible for public utilization via the CompChem database (www.compchem.net/ccdb).

### Docking computations

All investigated ANPs were docked into the binding pocket of VP35 employing AutoDock4.2.6 software [40]. In the current study, standard and expensive precision levels were employed to virtually screen the ANP database. Before conducting docking calculations, the PDB file of VP35 was transformed into PDBQT format [41]. The docking computations were performed employing the Lamarckian genetic algorithm (*GA*), using 50 runs for standard calculations and 250 runs for expensive computations. The energy evaluation (*eval*) values were modified to 5,000,000 and 25,000,000 for standard and expensive docking calculations, respectively. All other docking parameters were configured to their default settings. To cover the VP35 binding pocket, the grid center coordinates were adjusted to *x* = 6.056, *y* = 13.261, and *z* = 1.686. The dimensions of the grid box were established as 60 Å × 40 Å × 60 Å, with a grid spacing of 0.375 Å. The predicted poses were clustered utilizing an RMSD threshold of 1.0 Å. The representative pose was selected in accordance with the pose that exhibited the lowest docking score in the largest cluster.

### Molecular dynamics simulations (MDS)

MDS of the top-scored ANPs and the reference inhibitors (galidesivir and favipiravir) complexed with VP35 was performed utilizing AMBER20 software [42]. The specifics regarding the parameters of the MDS are outlined in the references [43–46]. For the structural parameterization, ANPs were treated by the general AMBER force field (GAFF2) [47]. Additionally, the VP35 was parameterized with the assistance of an AMBER force field 14SB [48]. The restricted electrostatic potential (RESP) approach was employed to determine the atomic charges of the examined inhibitors at the HF/6-31G^*^ level utilizing Gaussian09 software [49,50]. Each inhibitor-VP35 complex was neutralized through the insertion of Na^+^/Cl^−^ counterions using the LEAP module. The investigated inhibitor-VP35 complexes were immersed in the TIP3P water model in an octahedral box with dimensions of 12.0 Å. Thereafter, energy minimization for the solvated complexes was performed for 5,000 steps utilizing the steepest descent and conjugate gradient algorithms. The heating from 0 to 310 K was then conducted over a duration of 50 ps utilizing the NVT ensemble. Thereafter, all investigated complexes were equilibrated over 10 ns utilizing the NPT ensemble. Finally, the production stage for each complex was executed for 5, 100, and 250 ns. All MDS were accomplished on the GPU utilizing the CUDA version of PMEMD integrated with the AMBER20 package. BIOVIA Discovery Studio Visualizer was utilized to illustrate all molecular interactions [51].

### Binding affinity estimations

Binding energy (Δ*G*_binding_) for the identified potent ANPs in complex with VP35 was computed over the MDS utilizing the molecular mechanical/generalized Born surface area (MM/GBSA) approach [52]. The trajectories of each complex were collected from the MDS at intervals of 10 ps, and subsequently, all water molecules and counterions were eliminated prior to the MM/GBSA computations. The Δ*G*_binding_ was computed in the following manner.


$$\;{\Delta G}_{\text{binding}}=G_{\text{VP35}-\text{ANP }}-G_{\text{VP35}}\;-G_{\text{ANP}}$$ (1)

The *G* term can be derived as follows:


$$\;G={G_{\text{sol}}\;+E}_{\text{MM}}-TS$$ (2)

$$\;E_{\text{MM}}={E_{\text{ele}}\;+E}_{\text{int}}+E_{\text{vdW}}$$ (3)

$$\;G_{\text{sol}}={G_{\text{SA}}\;+G}_{\text{GB}}$$ (4)

$$\;G_{\text{SA}}=y.\text{SASA}+\text{b}$$ (5)

*E*_MM_ is molecular mechanics energy. *E*_MM_ comprises three components: the electrostatic energy (*E*_ele_), the internal energy (*E*_int_), which encompasses bond, dihedral, and angle energies, and the van der Waals energy (*E*_vdW_). The solvation-free energy (*G*_sol_) comprises both polar and nonpolar components. The polar participation (*G*_GB_) is determined using the GB model, while the nonpolar participation (*G*_SA_) is typically calculated through the solvent-accessible surface area (SASA) model. In the current study, the entropic contribution (*S*) in MM/GBSA assessment was disregarded due to its high computational cost [53,54].

### Drug-likeness and medicinal chemistry characteristics

The medicinal chemistry and drug-likeness features of the identified potent ANPs were predicted via the SwissADME web server [55]. In terms of drug-likeness properties, the subsequent criteria were examined: molecular weight (MW), number of hydrogen bond donors/ acceptors (HBD/HBA), logarithm of the octanol/water partition coefficient (Mlog *P*), and topological-polar surface area (TPSA). Furthermore, synthetic accessibility (SA) and pan-assay interference (PAINS) were estimated for the identified ANPs to confirm their medicinal chemistry characteristics [56]. To more effectively pinpoint possible drug candidates, ligand efficiency (LE) metrics were calculated as the docking score (DS) divided by the number of heavy atoms (NHA) [57,58].

### Pharmacokinetic and toxicity prediction

A majority of drug candidates do not succeed in the discovery phase when it comes to entering clinical trials, largely because of their ineffective pharmacokinetic (PK) characteristics. The PK characteristics (ADME: absorption, distribution, metabolism, and excretion) of the identified ANPs were estimated using the pkCSM web server [59]. Absorption was predicated upon water solubility (log*S*), while distribution was estimated based on permeability through the blood-brain barrier (BBB). The metabolism was evaluated according to CYP3A4/CYP2D6 substrates. A substrate for renal OCT2 was used to assess excretion. Ultimately, the toxicity was evaluated in accordance with the AMES toxicity assessment.

### DFT calculations

To examine the chemical reactivity of the identified ANPs, DFT calculations were performed on the last frame retrieved from the MDS. The identified ANPs were first geometrically optimized at the M06-2X/6–311 + G** level using the Gaussian09 software [50,60]. This level of theory was adopted as it aligns with methodologies commonly employed in related literature [61–63]. In order to understand the electrophilic and nucleophilic nature, the electrostatic potential (ESP) examination was conducted on all optimized ANPs. Within the framework of ESP analysis, molecular electrostatic potential (MEP) maps were evaluated utilizing an electron density contour of 0.002 au [64]. Frontier molecular orbitals (FMOs) theory was also applied to the identified ANPs, providing electronic insights. From this perspective, the energetic values and electronic patterns of the lowest unoccupied molecular orbital (LUMO) and the highest occupied molecular orbital (HOMO) were investigated. As a result, the energy gap (*E*_gap_) and the Fermi level (*E*_FL_) energies were determined using the *E*_HOMO_ and *E*_LUMO_ energy values, as demonstrated in the equations below:


$$E_{\text{gap}}=E_{\text{LUMO}}-E_{\text{HOMO}}$$ (6)

$$\;E_{FL}=E_{\text{HOMO}}+\;\frac{E_{\text{LUMO}}-E_{\text{HOMO}}\;}{\text{2}}$$ (7)

From the *E*_HOMO_ and *E*_LUMO_ values, further quantum mechanical (QM) descriptors were determined for the identified ANPs. Among QM descriptors, ionization potential (*IP*), electrophilicity index (*ω*), global hardness (*η*), electron affinity (*EA*), global softness (*S*), and chemical potential (*μ*) were computed as follows:


$$\;IP\approx -E_{HOMO}\;$$ (8)

$$\;EA\approx -E_{LUMO}\;$$ (9)

$$\;S=\;\frac{\text{1}}{\eta }$$ (10)

$$\mu =\frac{E_{LUMO}+E_{HOMO}}{\text{2}}$$ (11)

$$\eta =\frac{E_{LUMO}-E_{HOMO}}{\text{2}}$$ (12)

$$\;\omega =\frac{\mu^{\text{2}}}{\text{2}\eta }$$ (13)

## Results and discussion

### Virtual screening of ANP database

**Fig 2:**
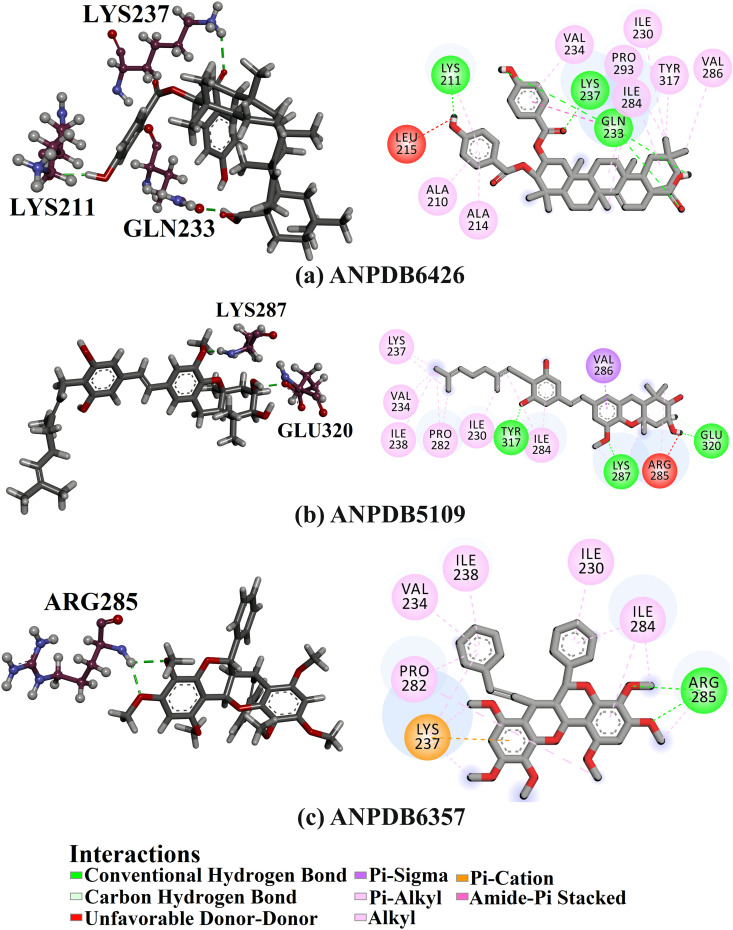
3D and 2D illustrations of the anticipated docking modes of (a) ANPDB6426, (b) ANPDB5109, and (c) ANPDB6357 within the VP35 binding pocket. 3D views show ligand (grey sticks) and key VP35 residues (ball and stick format), with hydrogen bonds (green dashed lines).

To hunt putative VP35 inhibitors derived from natural product sources, the ANP database, encompassing over 6,500 ANPs, was virtually screened using AutoDock4.2.6 software. To decrease the expenses and time of the *in-silico* computations, the investigated ANP database was first screened against VP35 using standard docking estimations with *eval *= 5,000,000 and *GA* = 50. Based on the standard docking scores, 371 ANPs showed docking scores <−7.0 kcal/mol towards VP35. The top-scored 371 ANPs from the standard docking computations were chosen and re-docked towards VP35 with expensive parameters (i.e., *eval *= 25,000,000 and *GA* = 250). The expensive docking scores are given in S1 Table. From S1 Table, 68 ANPs revealed docking scores <−8.0 kcal/mol towards VP35. Of note, −7.0 and −8.0 kcal/mol were opted as threshold values to narrow down the most potent VP35 inhibitors. The evaluated docking scores and the intramolecular H-bonds of the three most potent ANPs with VP35 are listed in Table 1. Additionally, Fig 2 depicts both the 3D and 2D binding patterns between these three ANPs with the VP35 binding pocket. What stands out regarding the binding interactions shown in Fig 2 is that a majority of the investigated ANPs revealed nearly the same docking poses, displaying a significant interaction with LYS237. It is important to highlight that these three ANPs were selected based on their assessed binding affinity over 250 ns, as evidenced in the MDS section.

**Table 1:** Evaluated docking scores and the intramolecular H-bonds of three potent ANPs with VP35 ^a^.

Compound Code/Name	Docking Score (kcal/mol)		Intramolecular H-bonds
	Standard	Expensive	
**ANPDB6426** ((4a*S*,6b*R*,10*R*,11*R*,12a*R*,14a*S*)-10,11-bis[(4-hydroxybenzoyl)oxy]-2,2,6b,9,9,12a,14a-heptamethyl-1,3,4,5,6a,7,8,8a,10,11,12,13,14,14b-tetradecahydropicene-4a-carboxylic acid)	−8.9	−9.1	LYS211 (2.14 Å), GLN233 (1.98 Å), LYS237 (2.35 Å)
**ANPDB5109** (Schweinfurthin B) (7-[2-[4-(3,7-dimethylocta-2,6-dienyl)-3,5-dihydroxyphenyl]ethenyl]-5-methoxy-1,1,4a-trimethyl-3,4,9,9a-tetrahydro-2H-xanthene-2,3-diol)	−8.7	−8.3	TYR317 (1.70 Å), GLU320 (1.84 Å), LYS287 (1.67 Å)
**ANPDB6357** (Dependensin) (1,3,4,8,10,11-Hexamethoxy-6-phenyl-7-(2-phenylethenyl)-6,6a,7,12a-tetrahydrochromeno[3,2-c]chromene)	−8.0	−8.3	ARG285 (2.25; 2.18 Å)

^a^ The ANPs were arranged based on the expensive docking evaluations.

ANPDB6426 unveiled the lowest docking score of −9.1 kcal/mol, forming three H-bonds with the essential residues of VP35. Examining the docking pose of ANPDB6426 revealed that the OH group engaged in forming two H-bonds with the CO of GLN233 (1.98 Å) and CO of LYS211 (2.14 Å). Moreover, the CO group contributed to establishing an H-bond with NH_3_ of LYS237 (2.35 Å) (Fig 2). As well, ANPDB6426 displayed an amide pi-stacked interaction with GLN233.

ANPDB5109, a natural compound made by bees from sap and buds found on plants and trees, demonstrated the second lowest docking score of −8.3 kcal/mol. Analyzing the docking pose of ANPDB5109 within the VP35 binding pocket disclosed that the two OH groups established two H-bonds with the OH of TYR317 (1.70 Å) and the carboxylate of GLU320 (1.84 Å). Besides, the OCH_3_ interacted with the NH of LYS287, forming an H-bond with a bond length of 1.67 Å. ANPDB5109 also demonstrated pi-sigma interaction with VAL286 (Fig 2).

ANPDB6357 is a dimeric flavonoid extracted from the root bark of a medicinal species found in Tanzania and demonstrates significant antimalarial effectiveness [65]. ANPDB6357 displayed a promising docking score toward VP35 with a value of −8.3 kcal/mol. Inspecting the docking pose of ANPDB6357 disclosed that the two OCH_3_ exhibited two H-bonds with the NH of ARG285 (2.25 and 2.18 Å). In addition, ANPDB6357 exhibited pi-cation interaction with LYS237 and a carbon H-bond with VAL283 (Fig 2).

### Molecular dynamics simulations (MDS)

**Fig 3:**
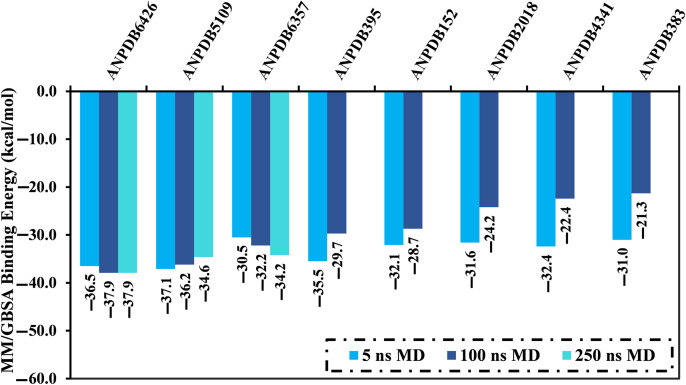
Evaluated MM/GBSA binding energies of the most potent eight ANPs with the VP35 over 5, 100, and 250 ns MDS.

To investigate the conformational steadiness in greater depth and to confirm the docking outcomes, MDS was performed on the most potent ANPs complexed with VP35. The top-scored 68 ANPs with docking scores <−8.0 kcal/mol against VP35 were advanced for MDS through 5 ns to lessen the *in-silico* cost. The corresponding MM/GBSA binding energies were estimated and are recorded in S2 Table. Depending on the data provided in S2 Table, only eight ANPs exhibited binding energies lower than −30.0 kcal/mol toward VP35. To obtain more reliable results, the eight potent ANPs bound to the VP35 underwent extended MDS lasting 100 ns, followed by evaluations of their binding energies (Fig 3). What can be deduced from Fig 3 is that only three ANPs, namely ANPDB6426, ANPDB5109, and ANPDB6357, demonstrated significant binding energies lower than −30.0 kcal/mol towards VP35. A threshold value of −30.0 kcal/mol was selected to shortlist the potent VP35 inhibitors. For the three most potent ANPs in complex with VP35, MDS were extended to 250 ns, and their binding energies were calculated (Fig 3). It is evident from Fig 3 that there were no significant variations observed in the calculated binding energies of the identified ANPs throughout the 100 and 250 ns MDS. It was found that ANPDB6426, ANPDB5109, and ANPDB6357 exhibited promising binding affinity towards VP35 over 250 ns MDS, with Δ*G*_binding_ values of −37.9, −34.6, and −34.2 kcal/mol, respectively. It is worth mentioning that the MM/GBSA approach reproduces the experimental relative binding affinities in good agreement with a *R*^2^ value > 0.89 [66,67]. The present findings shine a new light on the effectiveness of the identified ANPs as potential anti-MBV therapeutics.

**Fig 4:**
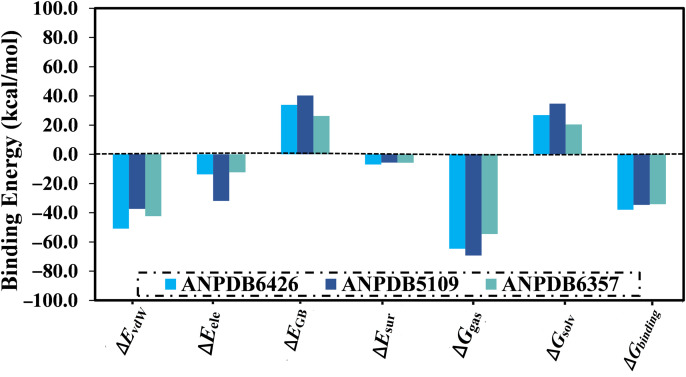
Binding energy components of ANPDB6426, ANPDB5109, and ANPDB6357 bound to VP35 over 250 ns MDS.

The Δ*G*_binding_ was also decomposed to understand the forces that dominated the interactions of ANPDB6426, ANPDB5109, and ANPDB6357 with VP35 (Fig 4). According to the energy decomposition analysis, the Δ*E*_vdW_ interactions predominantly influenced the Δ*G*_binding_ of ANPDB6426, ANPDB5109, and ANPDB6357, with mean values of −50.9, −37.4, and −42.3 kcal/mol, respectively. The Δ*E*_ele_ energies for ANPDB6426, ANPDB5109, and ANPDB6357 were also favorable, with average values of −13.8, −31.9, and −12.3, respectively, reflecting the strength of noncovalent electrostatic interactions between the identified ANPs and VP35.

**Fig 5:**
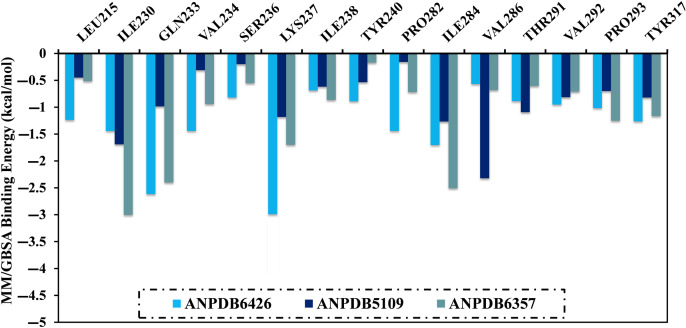
Per-residue energy decomposition of ANPDB6426, ANPDB5109, and ANPDB6357 bound to VP35 throughout 250 ns MDS.

A per-residue energy decomposition was fulfilled to inspect the contribution of the essential residues in the binding of ANPDB6426, ANPDB5109, and ANPDB6357 with VP35 (Fig 5). Only residues exhibiting Δ*G*_binding_ <−0.5 kcal/mol were considered in Fig 5. According to data listed in Fig 5, ILE230, GLN233, LYS237, and ILE284 showed significant roles in the interaction of ANPDB6426, ANPDB5109, and ANPDB6357 with VP35. For instance, the LYS237 displayed Δ*G*_binding_ values of −3.1, −1.2, and −1.7 kcal/mol for ANPDB6426-, ANPDB5109-, and ANPDB6357-VP35 complexes, respectively.

### Post-MD analyses

While molecular docking and MDS, along with binding energy evaluation, indicated the potentiality of identified ANPs as VP35 inhibitors, further analyses based on MDS would be necessary to inspect the structural and energetical stabilities of ANP-VP35 complexes. The evaluations of structural and energetic analyses comprised binding energy per frame, distance of center of mass (CoM), root-mean-square fluctuation (RMSF) and deviation (RMSD), solvent-accessible surface area (SASA), radius of gyration (Rg), and H-bond analysis.

#### Distance of CoM.

**Fig 6:**
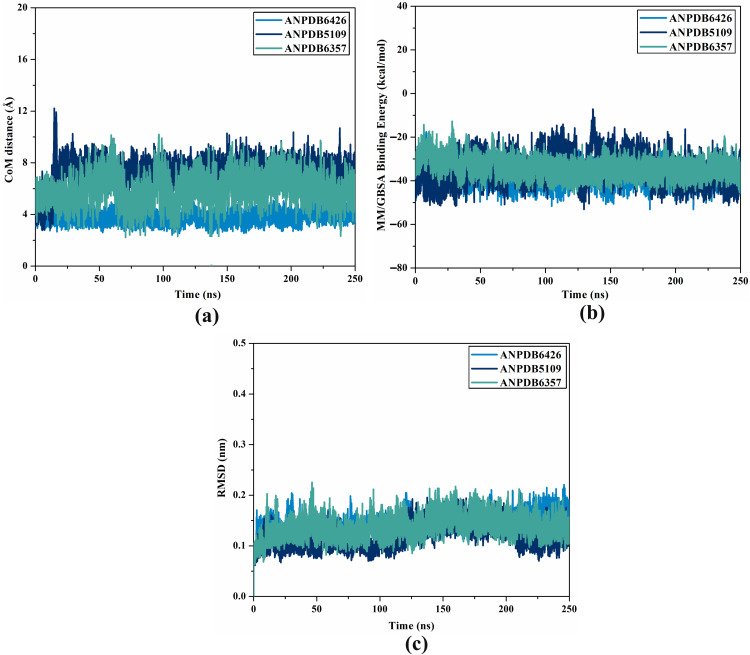
Plot of (a) Estimated distance of CoM, (b) binding affinity per-frame, and (c) RMSD of ANPDB6426 (light blue), ANPDB5109 (navy), and ANPDB6357 (cyan) bound to VP35 throughout 250 ns MDS.

The CoM distance between the ligand and a crucial residue would provide valuable understanding regarding the constancy of the inhibitor-receptor complex throughout MDS. Consequently, the CoM distance between ANPDB6426, ANPDB5109, and ANPDB6357 and LYS237 was measured and is given in Fig 6a. ANPDB6426-, ANPDB5109-, and ANPDB6357-VP35 complexes had narrow-fluctuating CoM distances, with mean values of 3.8, 7.0, and 5.9 Å, respectively. These findings revealed the comprehensive steadiness of the identified ANPs inside the VP35 binding pocket over 250 ns MDS.

#### Binding affinity per-frame.

To investigate the energetical stability of the ANP-VP35 complexes, the binding energy for each frame was assessed over 250 ns MDS (Fig 6b). According to Fig 6b, the general steadiness of the ANPDB6426, ANPDB5109, and ANPDB6357 bound to VP35 was noted over the MDS, exhibiting mean Δ*G*_binding_ values of −37.9, −34.6, and −34.2 kcal/mol, respectively. The most prominent outcome of this analysis is that all investigated complexes exhibited stability throughout the 250 ns MDS.

#### RMSD analysis.

The structural steadiness of the identified ANPs bound to the VP35 was gauged using RMSD analysis (Fig 6c). From Fig 6c, the mean RMSD values were observed to be 0.14, 0.12, and 0.14 nm for ANPDB6426, ANPDB5109, and ANPDB6357 bound to VP35, respectively. These results suggested that the identified ANPs were tightly bound to VP35 and did not impact the comprehensive topology of VP35.

#### Rg analysis.

**Fig 7:**
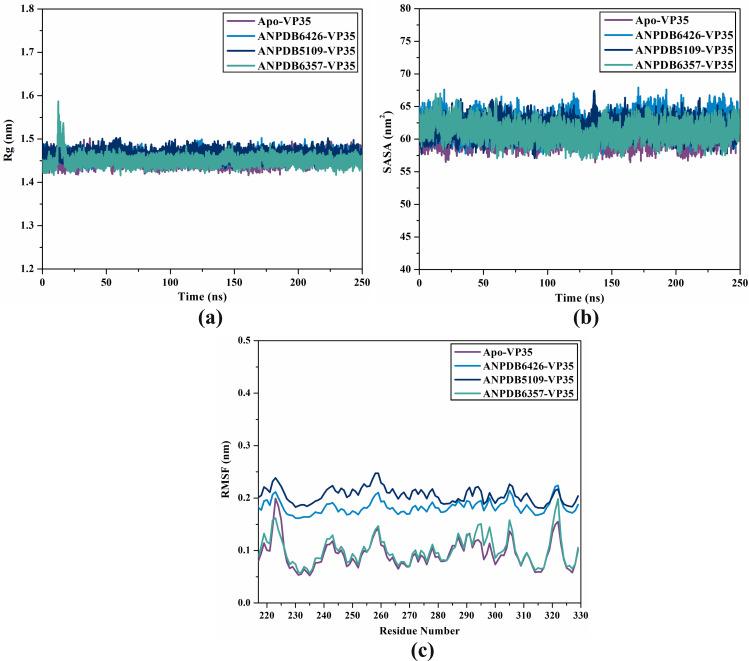
Plot of (a) Rg, (b) SASA, and (c) RMSF analyses for apo-VP35 (mauve), ANPDB6426-VP35 (light blue), ANPDB5109-VP35 (navy), and ANPDB6357-VP35 (cyan) throughout 250 ns MDS.

The Rg value serves as a physical metric to define the alterations in protein structure; proteins that exhibit considerable structural stability tend to possess greater compactness and, consequently, small Rg values. The ANPDB6426-, ANPDB5109-, and ANPDB6357-VP35 demonstrated lower average Rg values compared to apo-VP35 with mean Rg values of 1.46, 1.45, 1.44, and 1.43 nm, respectively (Fig 7a). These findings indicated that the binding of ANPDB6426, ANPDB5109, and ANPDB6357 considerably stabilized the VP35 structure.

#### SASA analysis.

Moreover, SASA indicates the regions of the receptor that are adequately accessible to interact with surrounding solvent molecules. SASA is fundamental in the examination of protein stability and folding, characterized as the hypothetical center of the solvent sphere that interacts with the surface of the molecule via vdW interactions [68]. The mean SASA values for apo-VP35, ANPDB6426-VP35, ANPDB5109-VP35, and ANPDB6357-VP35 were found to be 60.3, 62.2, 61.5, and 61.0 nm^2^, respectively (Fig 7b). As shown in Fig 7b, no significant alteration was detected in the SASA values as a result of ligand interactions.

#### RMSF analysis.

To assess the extent of displacement variation over MDS, RMSF for the backbone atoms of apo-VP35, ANPDB6426-VP35, ANPDB5109-VP35, and ANPDB6357-VP35 were measured over 250 ns MDS (Fig 7c). Amino acids with RMSF values greater than 0.25 nm are recognized as flexible regions for robust interactions [69]. From Fig 7c, the mean of RMSF values were 0.10, 0.18, 0.21, and 0.10 nm for apo-VP35, ANPDB6426-VP35, ANPDB5109-VP35, and ANPDB6357-VP35, respectively. According to these findings, the majority of the VP35 amino acids demonstrated high stability and rigid regions when bound to ANPDB6426, ANPDB5109, and ANPDB6357.

#### H-bond number.

**Fig 8:**
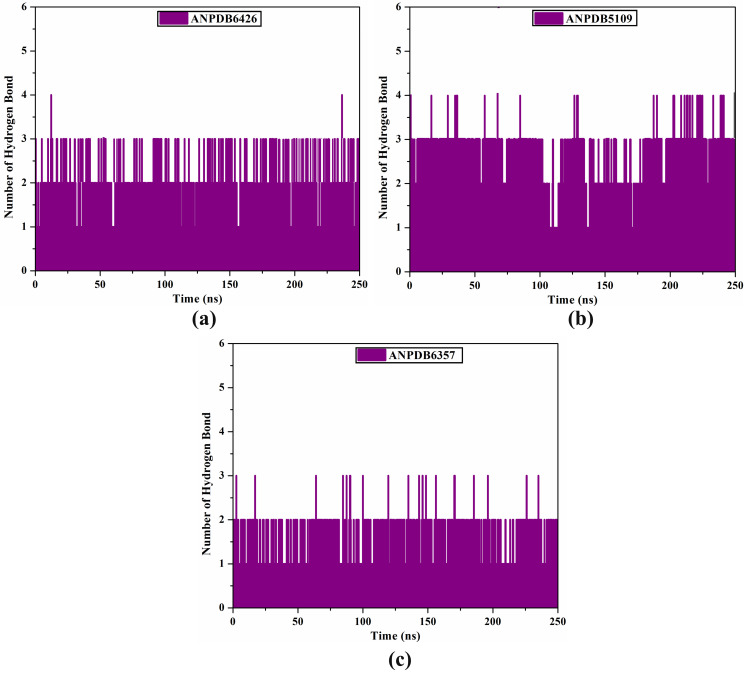
Number of H-bonds for (a) ANPDB6426, (b) ANPDB5109, and (c) ANPDB6357 bound to VP35 binding pocket over 250 ns MDS.

An important part of biomolecular stability is the H-bonding interaction; therefore, the number of H-bonds between the identified ANPs and the main residues of VP35 was estimated throughout 250 ns MDS (Fig 8). From Fig 8, the mean number of H-bonds was 3, 3, and 2 for ANPDB6426-, ANPDB5109-, and ANPDB6357-VP35 complexes, respectively. These results indicated good steadiness and inhibitory activity of the investigated ANPs and VP35 over 250 ns MDS.

### Drug-likeness and medicinal chemistry characteristics

Lipinski’s Rule of Five serves as a commonly employed initial tool for detecting the drug-like characteristics of a compound and determining its oral bioavailability [70,71]. The drug-likeness outcomes for all the identified ANPs are compiled in Table 2. According to Table 2, the MW of the identified ANPs ranged from 562.7 to 712.9 Da. For drugs that are active when taken orally, the Mlog *P* value should be less than 5.0 to achieve an ideal equilibrium between permeability and first-pass clearance. All the identified ANPs exhibited Mlog *P* values ≤ 5.0, with the exception of ANPDB6426, which had a value of 6.2. ANPDB6426 truly had Mlog *P* > 5; it falls outside Lipinski’s optimal range, potentially leading to high lipophilicity, low solubility, and absorption issues. This highlights the necessity to optimize the hydrophilicity profile of ANPDB6426 before it undergoes preclinical or clinical assessment. The number of rotatable bonds (N_rot_) and TPSA influence the passive transport of molecules, enabling the prediction of absorption characteristics of medications. The TPSA of the identified ANPs ranged from 73.8 to 130.4 Å², while the N_rot_ was found to be ≤ 10 (Table 2). The predicted TPSA values and N_rot_ indicated that the identified ANPs demonstrated high polarity, which revealed their ability to cross cell membranes. The number of HBD for the identified ANPs was < 5. On the other hand, all identified ANPs revealed a number of HBA less than 10, revealing good permeability and absorption characteristics. Additionally, the identified ANPs demonstrated good bioavailability scores (BS) with a value of 0.55, except ANPDB6426 unveiled BS with a value of 0.56. The identified ANPs successfully met the PAINS criteria, as no alerts of the PAINS pattern were detected for these ANPs, reinforcing the specificity of the identified ANPs against VP35. The SA score is fundamentally connected to how easily the identified ANPs can be synthesized. The SA score varies from one to ten, with one signifying extremely easy synthesis of the compound and ten signifying extremely difficult synthesis. ANPDB6426, ANPDB5109, and ANPDB6357 demonstrated SA scores of 7.05, 6.09, and 5.82, respectively. Observably, an SA score of ANPDB6426 is slightly above 7, reflecting possible difficulty in its synthesis using conventional methods; however, non-standard or multistep routes may be a choice for the synthesis of ANPDB6426 [72]. According to Table 2, it is evident that ANPDB6426, ANPDB5109, and ANPDB6357 exhibited LE values of −0.18, −0.20, and −0.19 kcal/mol/heavy atom, respectively. These findings demonstrated the favorable ligand efficiency of the identified ANPs, endorsing them as potential anti-MBV drug candidates.

**Table 2:** Drug-likeness, medicinal chemistry, and LE features of the identified ANPs as prospective anti-MBV drug candidates.

Compound Name/Code	MW	Mlog *P*	TPSA	N_rot_	HBD	HBA	PAINS	BS	SA	LE
ANPDB6426	712.9	6.2	130.4	7	3	8	0	0.56	7.05	−0.18
ANPDB5109	562.7	5.0	99.4	8	4	6	0	0.55	6.09	−0.20
ANPDB6357	596.8	3.3	73.8	9	0	8	0	0.55	5.82	−0.19

### ADMET characteristics

The primary causes for the failure of drugs during clinical trials are their undesirable pharmacokinetic characteristics and inadmissible toxicity. There is an immediate scientific necessity to choose suitable drug candidates that exhibit an ideal equilibrium between potency and ADMET [73]. The ADMET characteristics of the identified ANPs are compiled in Table 3. Compounds with log*S* values higher than –6.0 are generally considered to have acceptable solubility for oral administration, while those with log*S* values between –4.0 and –2.0 demonstrate good solubility. Concerning the absorption characteristic, the ANPDB6426, ANPDB5109, and ANPDB6357 demonstrated water solubility (log*S*) with values of –3.2, –5.0, and –4.8 mol/L, respectively, indicating their good solubility. With regards to distribution characteristics, the logBB values for ANPDB6426, ANPDB5109, and ANPDB6357 were –1.0, –0.9, and –1.1, respectively. A compound exhibiting a logBB value exceeding 0.3 can easily cross the blood-brain barrier, while a compound with a logBB value lower than –1.0 is not well distributed to the brain. The obtained values of log BB of the identified ANPs indicated low to moderate brain penetration, which may be favorable for systemic antiviral activity with limited central nervous system side effects. Regarding metabolism, the identified ANPs were known as substrates for CYP3A4. With regard to excretion, all the identified ANPs were recognized as non-substrates for renal OCT2. In terms of toxicity, none of the identified ANPs exhibited AMES toxicity (Table 3).

**Table 3:** The ADMET features of the identified ANPs as potential anti-MBV drug candidates.

Compound Name/Code	Absorption	Distribution	Metabolism	Excretion	Toxicity
	log*S*	logBB	CYP3A4 Substrate	Renal OCT2	AMES Test
ANPDB6426	–3.2	–1.0	Yes	No	No
ANPDB5109	–5.0	–0.9	Yes	No	No
ANPDB6357	–4.8	–1.1	Yes	No	No

### QM computations

**Fig 9:**
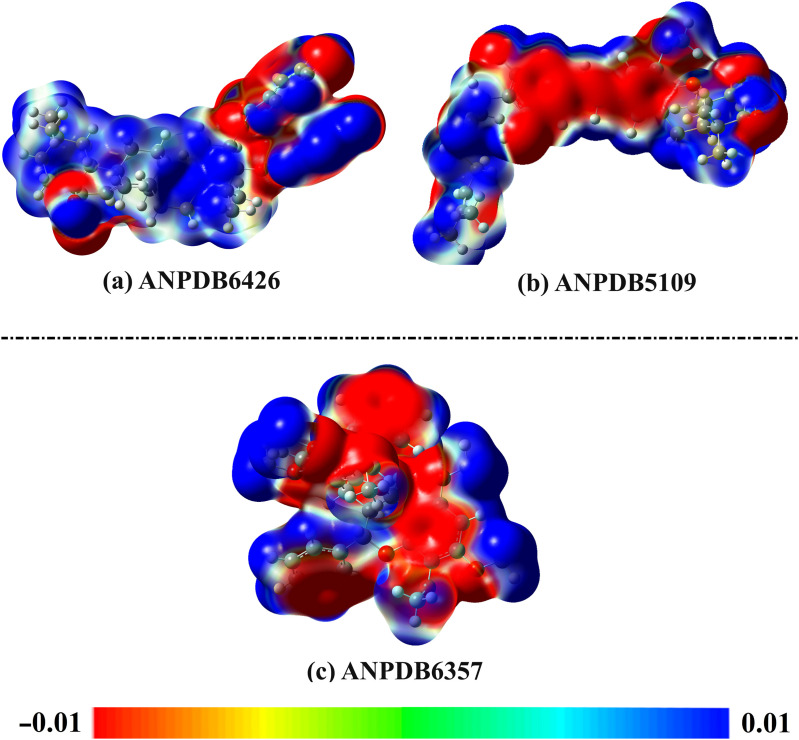
MEP maps of the last frame of (a) ANPDB6426, (b) ANPDB5109, and (c) ANPDB6357, with the color scale from −0.01 (red) to +0.01 (blue) au.

Based on the geometry optimization of the final frame of the identified ANPs, the ESP surface was examined by creating maps of MEP (Fig 9). Based on the MEP maps, multiple sites of charge accumulation (i.e., red zones) were identified above the N and O atoms of the identified ANPs, signifying their nucleophilic nature. Additionally, significant electron depletion sites (i.e., blue sites) were observed for H-atoms of the identified ANPs, indicating their electrophilic nature. Upon these observations, the identified ANPs revealed the ability to establish H-bonds inside the VP35 binding pocket.

**Fig 10:**
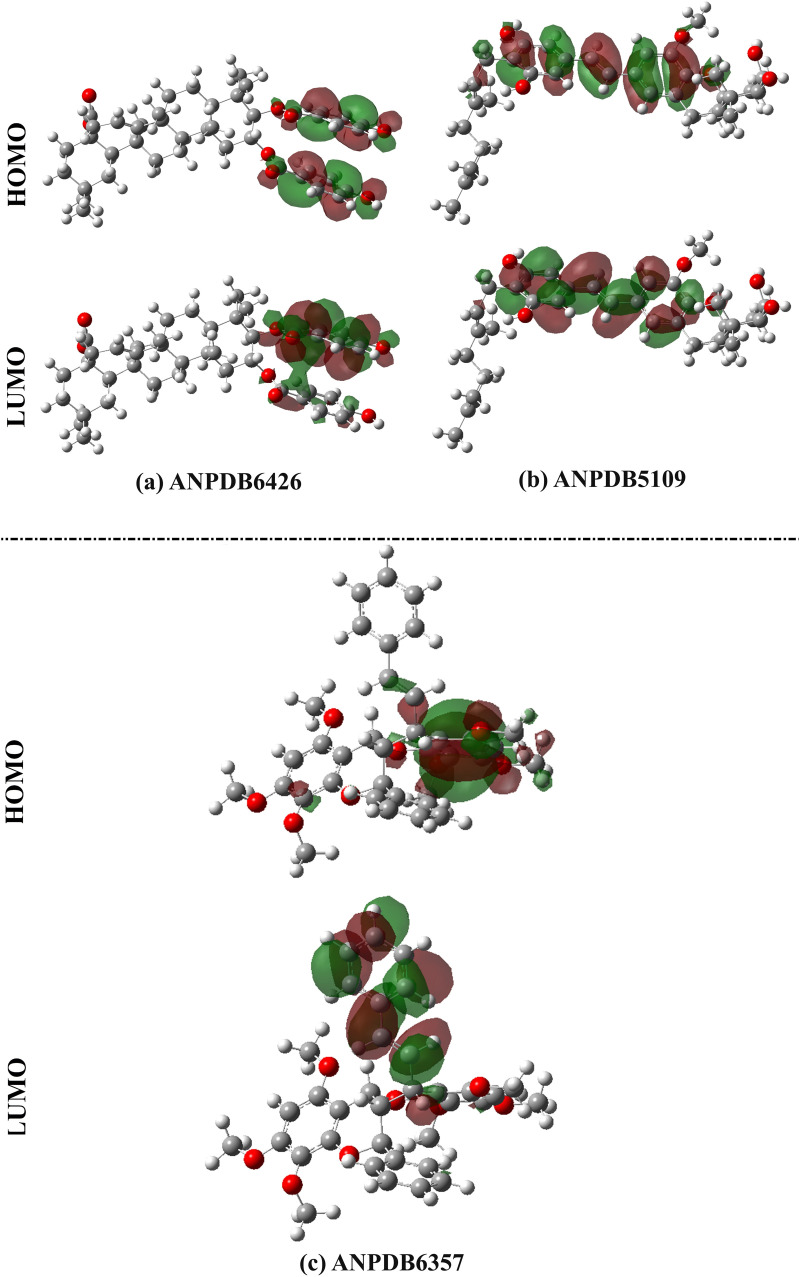
The HOMO and LUMO distributions of (a) ANPDB6426, (b) ANPDB5109, and (c) ANPDB6357.

To adequately illustrate the electronic characteristics of the identified ANPs, the frontier molecular orbitals (FMOs) theory was utilized. According to FMOs, the energy values of HOMO and LUMO (i.e., *E*_HOMO_ and *E*_LUMO_, respectively) were calculated. As well, the energy gap (*E*_gap_) and Fermi level (*E*_FL_) were further computed (Table 4). In the core of the FMOs theory, graphical depictions of the HOMO and LUMO for the identified ANPs were generated and are illustrated in Fig 10.

**Table 4:** Estimated QM parameters of the optimized ANPDB6426, ANPDB5109, and ANPDB6357.

Compound Name/Code	*E*_HOMO_ (eV)	*E*_LUMO_ (eV)	*E*_gap_ (eV)	*E*_FL_ (eV)	*IP* (eV)	*EA*(eV)	*µ* (eV)	*η* (eV)	*S* (eV^ − 1^)	*ω* (eV)
ANPDB6426	–7.7	–0.4	7.3	–4.1	7.7	0.4	–4.1	3.7	0.3	2.2
ANPDB5109	–6.6	–0.6	6.0	–3.6	6.6	0.6	–3.6	3.0	0.3	2.2
ANPDB6357	–6.9	–0.1	6.8	–3.5	6.9	0.1	–3.5	3.4	0.3	1.8

As demonstrated in Fig 10, the HOMO levels were predominantly located over the nucleophilic regions (i.e., N and O atoms) of the identified ANPs, while the LUMO orbitals were focused on the electrophilic areas (i.e., H and C atoms) of the examined ANPs. By analyzing data in Table 4, the *E*_HOMO_/*E*_LUMO_ values were found to be –7.7/–0.4, –6.6/–0.6, and –6.9/–0.1 eV for ANPDB6426, ANPDB5109, and ANPDB6357, respectively. As well, ANPDB6426, ANPDB5109, and ANPDB6357 unveiled low *E*_gap_ with values of 7.3, 6.0, and 6.8 eV, respectively. It is important to highlight that the low *E*_gap_ values revealed the significant chemical reactivity of the identified ANPs. The identified ANPs showed promising *E*_FL_ with values of –4.1, –3.6, and –3.5, respectively.

In order to obtain more dependable insights into electronic characteristics, global reactivity descriptors were computed for the identified ANPs (Table 4). From Table 4, ANPDB6426, ANPDB5109, and ANPDB6357 exhibited *IP* with values in the range of 6.6 to 7.7 eV. Additionally, the *η* values for ANPDB6426, ANPDB5109, and ANPDB6357 were 3.7, 3.0, and 3.4 eV, respectively. To sum up, the identified ANPs displayed significant chemical reactivity based on the calculated global descriptors.

### Comparison between the identified ANPs and reference drugs

Galidesivir is a type of adenosine analog that demonstrated potential effectiveness against the Zaire Ebola virus. Galidesivir has recently undergone clinical studies as a promising anti-MBV medication [74]. Favipiravir is a broad-spectrum antiviral drug originally developed for the treatment of influenza [75]. Favipiravir acts as a selective inhibitor of viral RNA-dependent RNA polymerase (RdRp), thereby disrupting viral replication [76]. Due to its mechanism of action, favipiravir has demonstrated *in-vitro* and *in-vivo* activity against a wide range of RNA viruses, including Ebola, Lassa, Zika, and Marburg viruses [77]. For this reason, galidesivir and favipiravir were employed as reference inhibitors in the present investigation. To evaluate the potentiality of the identified ANPs, the docking modes and scores of ANPDB6426, ANPDB5109, and ANPDB6357 were compared to galidesivir and favipiravir bound to VP35. Consequently, docking evaluations and MDS, along with binding energy estimations, were performed for galidesivir and favipiravir complexed with VP35 (S1 Fig and S3 Table). As evidenced by the data presented in S1 Fig and S3 Table, ANPDB6426, ANPDB5109, ANPDB6357, galidesivir, and favipiravir exhibited similar docking poses with VP35, demonstrating H-bond interactions with essential residues within the binding pocket of VP35. More precisely, inspecting the docking mode of galidesivir inside the VP35 binding pocket unveiled that OH, NH_3,_ and NH_2_ of the galidesivir established three H-bonds with OH of TYR317 (1.89 Å) and two CO groups of GLN233 (2.66 and 1.77 Å). Analyzing the docking pose of favipiravir inside the VP35 binding pocket revealed that the CO and NH_2_ of favipiravir formed two H-bonds with NH_3_ of LYS237 (1.72 Å) and CO of VAL234 (2.11 Å) (S1 Fig). Interestingly, ANPDB6426, ANPDB5109, and ANPDB6357 unveiled superior docking scores compared to galidesivir and favipiravir, with values of −9.1, −8.3, −8.3, −6.1, and −4.9 kcal/mol, respectively. Of note, the greater docking scores of identified ANPs compared to reference inhibitors may be imputed to other interactions, like van der Waals and hydrophobic interactions with the proximal residues within the VP35 binding pocket.

In the realm of 250 ns MDS and MM/GBSA binding energy computation, the mean Δ*G*_binding_ for galidesivir and favipiravir with VP35 were only −16.2 and −13.8 kcal/mol, respectively, in comparison with −37.9, −34.6, and −34.2 kcal/mol for ANPDB6426, ANPDB5109, and ANPDB6357, respectively. In a similar manner to the identified ANPs, the Δ*G*_binding_ of galidesivir and favipiravir with VP35 was dominated by *E*_vdW_ interactions, with a mean value of −13.0 and −12.2 kcal/mol, respectively. Based on the data comparison, it was observed that the Δ*G*_binding_ of the identified ANPs was approximately two times lower than galidesivir and favipiravir.

## Conclusion

MBV is a highly pathogenic agent responsible for hemorrhagic fever that causes high mortality rates. Currently, no medication has demonstrated efficacy in preventing the illness resulting from the MBV. VP35 is an attractive druggable target for hunting therapeutic agents to combat MBV owing to its vital role in viral reproduction and in circumventing the immune response of the host. Herein, the ANP database, encompassing over 6,500 ANPs, was virtually screened toward VP35 using *in-silico* computations. Upon *in-silico* computations, ANPDB6426, ANPDB5109, and ANPDB6357 displayed promising binding energies over 250 ns MDS with Δ*G*_binding_ values of −37.9, −34.6, and −34.2, respectively. The post-MD analyses confirmed the steadiness of the identified ANPs inside the VP35 binding pocket over 250 ns MDS. Upon the drug-likeness features, the identified ANPs unveiled favorable oral bioavailability properties. ADME characteristics of the identified ANPs fell within a favorable range, and the oral toxicity levels of these compounds were demonstrated to be safe. Quantum mechanical calculations were also performed, and their results revealed the high chemical reactivity of the identified ANPs. In comparison with galidesivir and favipiravir, reference inhibitors, the computed Δ*G*_binding_ of the identified ANPs with VP35 was approximately two times lower than galidesivir and favipiravir. These findings indicated that further *in-vitro*/*in-vivo* studies on the identified ANPs may yield putative VP35 inhibitors capable of counteracting MBV effects.

## Supporting information

S1 Fig 2D illustrations of the anticipated docking poses of (a) ANPDB6426, (b) ANPDB5109, (c) ANPDB6357, (d) galidesivir, and (e) favipiravir within the VP35 active site. (DOCX)

S1 Table The anticipated standard and expensive docking scores (in kcal/mol) for the top 371 NPs towards VP35 active site. (DOCX)

S2 Table Estimated standard and expensive docking scores and MM/GBSA binding energies (in kcal/mol) over 5 ns MD simulations of the promising 68 NPs towards VP35 active site. (DOCX)

S3 Table Computed docking score, conventional H-bonds, and Δ*G*_binding_ components over 250 ns MDS for ANPDB6426, ANPDB5109, ANPDB6357, and galidesivir bound to VP35. (DOCX)
