# Supplementary material for: Exploration of African natural products as VP35 inhibitors to combat Marburg virus infection: Molecular docking, molecular dynamics, and quantum mechanical computations
Source: PLoS One. 2025 Oct 24;20(10):e0334160. doi: 10.1371/journal.pone.0334160 (PMC12551841; doi:10.1371/journal.pone.0334160)
Supplement: S1 Fig — (DOCX) [file pone.0334160.s001.docx]

**
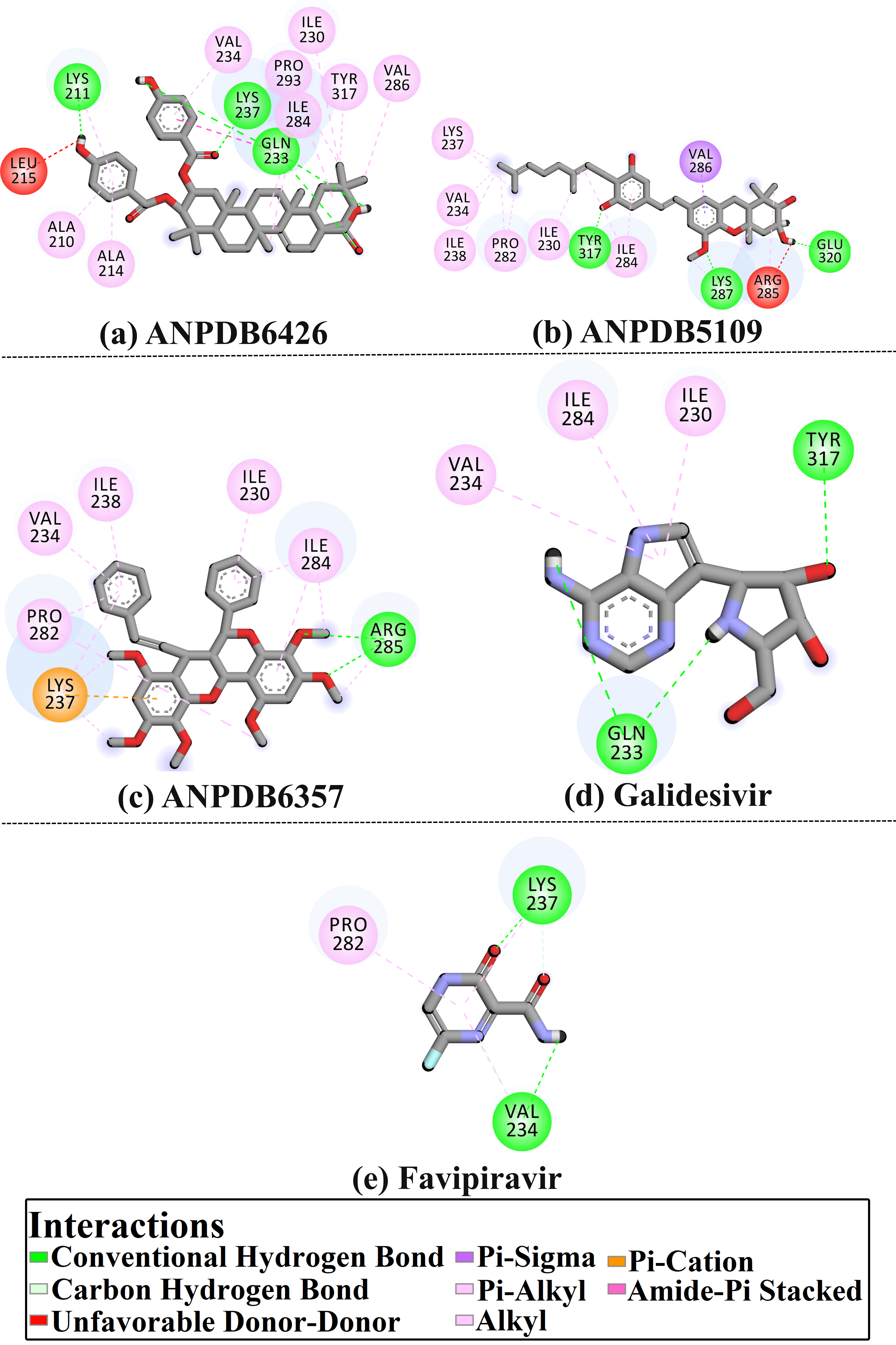
**

**S1 Fig.** 2D Illustrations of the anticipated docking poses of (a) ANPDB6426, (b) ANPDB5109, (c) ANPDB6357, (d) galidesivir, and (e) favipiravir within the VP35 active site.
