# Supplementary material for: Exploration of African natural products as VP35 inhibitors to combat Marburg virus infection: Molecular docking, molecular dynamics, and quantum mechanical computations
Source: PLoS One. 2025 Oct 24;20(10):e0334160. doi: 10.1371/journal.pone.0334160 (PMC12551841; doi:10.1371/journal.pone.0334160)
Supplement: S1 Table — (DOCX) [file pone.0334160.s002.docx]

**S1 Table.** The anticipated standard and expensive docking scores (in kcal/mol) for the top 371 NPs towards VP35 active site ^a^.

| No. | **Compound Code** | **Docking Score (kcal/mol)** | | **No**. | **Compound Code** | **Docking Score (kcal/mol)** | |
| --- | --- | --- | --- | --- | --- | --- | --- |
|  |  | **Standard** | **Expensive** |  |  | **Standard** | **Expensive** |
| 1 | ANPDB639 | −12.3 | −12.7 | 45 | ANPDB1867 | –8.1 | −8.1 |
| 2 | ANPDB5458 | −8.1 | −9.3 | 46 | ANPDB5461 | –8.1 | −8.1 |
| 3 | ANPDB3468 | −8.0 | −9.2 | 47 | ANPDB152 | –7.8 | −8.1 |
| 4 | ANPDB6426 | –9.0 | −9.1 | 48 | ANPDB6045 | –8.1 | −8.1 |
| 5 | ANPDB5629 | –9.0 | −9.0 | 49 | ANPDB2860 | –8.1 | −8.1 |
| 6 | ANPDB2018 | –7.9 | −9.0 | 50 | ANPDB5603 | –8.1 | −8.1 |
| 7 | ANPDB4294 | –7.5 | −9.0 | 51 | ANPDB1050 | –8.0 | −8.1 |
| 8 | ANPDB5459 | –8.9 | −9.0 | 52 | ANPDB1662 | –7.9 | −8.1 |
| 9 | ANPDB6433 | –7.8 | −8.9 | 53 | ANPDB851 | –8.1 | −8.1 |
| 10 | ANPDB935 | –7.5 | −8.9 | 54 | ANPDB5386 | –8.0 | −8.1 |
| 11 | ANPDB783 | –8.4 | −8.7 | 55 | ANPDB874 | –8.1 | −8.1 |
| 12 | ANPDB465 | –8.7 | −8.7 | 56 | ANPDB395 | –8.0 | −8.1 |
| 13 | ANPDB703 | –8.4 | −8.7 | 57 | ANPDB4341 | –8.1 | −8.1 |
| 14 | ANPDB1783 | –8.6 | −8.7 | 58 | ANPDB773 | –8.1 | −8.1 |
| 15 | ANPDB2343 | –8.6 | −8.7 | 59 | ANPDB1922 | –7.9 | −8.1 |
| 16 | ANPDB6362 | –8.6 | −8.6 | 60 | ANPDB872 | –8.1 | −8.1 |
| 17 | ANPDB3245 | –7.4 | −8.5 | 61 | ANPDB6474 | –8.1 | −8.1 |
| 18 | ANPDB1896 | –8.4 | −8.5 | 62 | ANPDB6369 | –7.9 | −8.1 |
| 19 | ANPDB383 | –8.4 | −8.5 | 63 | ANPDB3882 | –8.0 | −8.1 |
| 20 | ANPDB841 | –8.3 | −8.5 | 64 | ANPDB5053 | –7.7 | −8.1 |
| 21 | ANPDB6371 | –7.5 | −8.4 | 65 | ANPDB4740 | –7.9 | −8.1 |
| 22 | ANPDB6221 | –8.4 | −8.4 | 66 | ANPDB6326 | –8.0 | −8.1 |
| 23 | ANPDB1660 | –8.4 | −8.4 | 67 | ANPDB6039 | –8.0 | −8.1 |
| 24 | ANPDB375 | –8.4 | −8.4 | 68 | ANPDB871 | –8.0 | −8.1 |
| 25 | ANPDB6425 | –8.4 | −8.4 | 69 | ANPDB6013 | –8.0 | −8.1 |
| 26 | ANPDB648 | –8.4 | −8.4 | 70 | ANPDB5398 | –7.9 | −8.0 |
| 27 | ANPDB3364 | –8.3 | −8.4 | 71 | ANPDB3883 | –7.4 | −8.0 |
| 28 | ANPDB780 | –8.4 | −8.4 | 72 | ANPDB6206 | –7.4 | −8.0 |
| 29 | ANPDB643 | –8.3 | −8.3 | 73 | ANPDB1828 | –7.7 | −8.0 |
| 30 | ANPDB707 | –8.3 | −8.3 | 74 | ANPDB5349 | –8.0 | −8.0 |
| 31 | ANPDB5109 | –8.7 | −8.3 | 75 | ANPDB774 | –8.0 | −8.0 |
| 32 | ANPDB6357 | –8.0 | −8.3 | 76 | ANPDB2995 | –7.9 | −7.9 |
| 33 | ANPDB771 | –8.3 | −8.3 | 77 | ANPDB4989 | –7.8 | −7.9 |
| 34 | ANPDB2862 | –8.3 | −8.3 | 78 | ANPDB6421 | –7.2 | −7.9 |
| 35 | ANPDB405 | –8.3 | −8.3 | 79 | ANPDB6432 | –8.0 | −7.9 |
| 36 | ANPDB3881 | –8.2 | −8.3 | 80 | ANPDB1904 | –7.8 | −7.9 |
| 37 | ANPDB1919 | –8.2 | −8.2 | 81 | ANPDB2509 | –7.8 | −7.9 |
| 38 | ANPDB1837 | –8.0 | −8.2 | 82 | ANPDB5465 | –7.9 | −7.9 |
| 39 | ANPDB378 | –8.2 | −8.2 | 83 | ANPDB6014 | –7.9 | −7.9 |
| 40 | ANPDB840 | –8.1 | −8.2 | 84 | ANPDB6434 | –7.2 | −7.9 |
| 41 | ANPDB4293 | –8.1 | −8.2 | 85 | ANPDB151 | –7.8 | −7.9 |
| 42 | ANPDB6041 | –8.2 | −8.1 | 86 | ANPDB2908 | –7.7 | −7.9 |
| 43 | ANPDB6046 | –8.1 | −8.1 | 87 | ANPDB5462 | –7.8 | −7.9 |
| 44 | ANPDB2245 | –8.0 | −8.1 | 88 | ANPDB2623 | −7.9 | −7.9 |

**S1 Table.** *Continued*.

| No. | **Compound Code** | **Docking Score (kcal/mol)** | | **No**. | **Compound**  **Code** | **Docking Score (kcal/mol)** | |
| --- | --- | --- | --- | --- | --- | --- | --- |
|  |  | **Standard** | **Expensive** |  |  | **Standard** | **Expensive** |
| 89 | ANPDB2990 | −7.9 | −7.9 | 136 | ANPDB5443 | −7.7 | −7.7 |
| 90 | ANPDB5629 | −9.0 | −7.9 | 137 | ANPDB2268 | −7.5 | −7.7 |
| 91 | ANPDB3030 | −7.9 | −7.9 | 138 | ANPDB3039 | −7.7 | −7.7 |
| 92 | ANPDB376 | −7.9 | −7.9 | 139 | ANPDB4053 | −7.7 | −7.7 |
| 93 | ANPDB1661 | −7.8 | −7.8 | 140 | ANPDB611 | −7.3 | −7.7 |
| 94 | ANPDB1819 | −7.4 | −7.8 | 141 | ANPDB3439 | −7.5 | −7.6 |
| 95 | ANPDB436 | −7.8 | −7.8 | 142 | ANPDB5672 | −7.8 | −7.6 |
| 96 | ANPDB4968 | −7.8 | −7.8 | 143 | ANPDB6027 | −7.2 | −7.6 |
| 97 | ANPDB5537 | −7.2 | −7.8 | 144 | ANPDB6326 | −8.0 | −7.6 |
| 98 | ANPDB382 | −7.8 | −7.8 | 145 | ANPDB5599 | −7.1 | −7.6 |
| 99 | ANPDB5664 | −7.1 | −7.8 | 146 | ANPDB5942 | −7.1 | −7.6 |
| 100 | ANPDB5743 | −7.6 | −7.8 | 147 | ANPDB2601 | −7.6 | −7.6 |
| 101 | ANPDB5598 | −7.2 | −7.8 | 148 | ANPDB3062 | −7.6 | −7.6 |
| 102 | ANPDB6034 | −7.6 | −7.8 | 149 | ANPDB5603 | −8.1 | −7.6 |
| 103 | ANPDB4647 | −7.8 | −7.8 | 150 | ANPDB4629 | −7.5 | −7.6 |
| 104 | ANPDB1905 | −7.6 | −7.8 | 151 | ANPDB5358 | −7.4 | −7.6 |
| 105 | ANPDB1930 | −7.8 | −7.8 | 152 | ANPDB1060 | −7.5 | −7.6 |
| 106 | ANPDB1952 | −7.8 | −7.8 | 153 | ANPDB2239 | −7.4 | −7.6 |
| 107 | ANPDB290 | −7.7 | −7.8 | 154 | ANPDB4999 | −7.7 | −7.6 |
| 108 | ANPDB377 | −7.8 | −7.8 | 155 | ANPDB631 | −7.1 | −7.6 |
| 109 | ANPDB5670 | −7.8 | −7.8 | 156 | ANPDB1855 | −7.4 | −7.6 |
| 110 | ANPDB6369 | −7.9 | −7.8 | 157 | ANPDB1917 | −7.1 | −7.6 |
| 111 | ANPDB2052 | −7.1 | −7.8 | 158 | ANPDB2205 | −7.2 | −7.6 |
| 112 | ANPDB3884 | −7.5 | −7.8 | 159 | ANPDB2309 | −7.3 | −7.6 |
| 113 | ANPDB1055 | −7.5 | −7.8 | 160 | ANPDB1664 | −7.5 | −7.6 |
| 114 | ANPDB1627 | −7.4 | −7.8 | 161 | ANPDB2704 | −7.4 | −7.6 |
| 115 | ANPDB1903 | −7.7 | −7.8 | 162 | ANPDB3247 | −7.6 | −7.6 |
| 116 | ANPDB398 | −7.8 | −7.8 | 163 | ANPDB430 | −7.6 | −7.6 |
| 117 | ANPDB1842 | −7.8 | −7.8 | 164 | ANPDB153 | −7.5 | −7.6 |
| 118 | ANPDB2625 | −7.8 | −7.8 | 165 | ANPDB2732 | −7.3 | −7.6 |
| 119 | ANPDB3345 | −7.7 | −7.8 | 166 | ANPDB757 | −7.1 | −7.6 |
| 120 | ANPDB1777 | −7.7 | −7.8 | 167 | ANPDB3033 | −7.4 | −7.5 |
| 121 | ANPDB782 | −7.4 | −7.8 | 168 | ANPDB4116 | −7.3 | −7.5 |
| 122 | ANPDB2508 | −7.7 | −7.7 | 169 | ANPDB1238 | −8.0 | −7.5 |
| 123 | ANPDB3363 | −7.3 | −7.7 | 170 | ANPDB6206 | −7.4 | −7.5 |
| 124 | ANPDB6039 | −8.0 | −7.7 | 171 | ANPDB247 | −7.4 | −7.5 |
| 125 | ANPDB6321 | −7.1 | −7.7 | 172 | ANPDB3437 | −7.4 | −7.5 |
| 126 | ANPDB3119 | −7.6 | −7.7 | 173 | ANPDB5462 | −7.8 | −7.5 |
| 127 | ANPDB2964 | −7.7 | −7.7 | 174 | ANPDB798 | −7.4 | −7.5 |
| 128 | ANPDB4970 | −7.7 | −7.7 | 175 | ANPDB1690 | −7.3 | −7.5 |
| 129 | ANPDB3436 | −7.7 | −7.7 | 176 | ANPDB1844 | −7.5 | −7.5 |
| 130 | ANPDB2295 | −7.7 | −7.7 | 177 | ANPDB2863 | −7.5 | −7.5 |
| 131 | ANPDB2858 | −7.7 | −7.7 | 178 | ANPDB3987 | −7.5 | −7.5 |
| 132 | ANPDB374 | −7.7 | −7.7 | 179 | ANPDB4682 | −7.5 | −7.5 |
| 133 | ANPDB773 | −8.1 | −7.7 | 180 | ANPDB6329 | −7.6 | −7.5 |
| 134 | ANPDB778 | −7.5 | −7.7 | 181 | ANPDB2241 | −7.4 | −7.5 |
| 135 | ANPDB4627 | −7.7 | −7.7 | 182 | ANPDB3190 | −7.5 | −7.5 |

**S1 Table.** *Continued*.

| No. | **Compound Code** | **Docking Score (kcal/mol)** | | **No**. | **Compound**  **Code** | **Docking Score (kcal/mol)** | |
| --- | --- | --- | --- | --- | --- | --- | --- |
|  |  | **Standard** | **Expensive** |  |  | **Standard** | **Expensive** |
| 183 | ANPDB840 | −8.1 | −7.5 | 230 | ANPDB5880 | −7.1 | −7.3 |
| 184 | ANPDB3206 | −7.3 | −7.5 | 231 | ANPDB1036 | −7.3 | −7.3 |
| 185 | ANPDB6035 | −7.8 | −7.5 | 232 | ANPDB1481 | −7.2 | −7.3 |
| 186 | ANPDB1040 | −7.4 | −7.5 | 233 | ANPDB2179 | −7.3 | −7.3 |
| 187 | ANPDB2703 | −7.4 | −7.5 | 234 | ANPDB2865 | −7.3 | −7.3 |
| 188 | ANPDB75 | −7.3 | −7.5 | 235 | ANPDB2982 | −7.3 | −7.3 |
| 189 | ANPDB610 | −7.1 | −7.5 | 236 | ANPDB1691 | −7.3 | −7.3 |
| 190 | ANPDB6221 | −8.4 | −7.5 | 237 | ANPDB1916 | −7.2 | −7.3 |
| 191 | ANPDB6334 | −7.5 | −7.5 | 238 | ANPDB2690 | −7.3 | −7.3 |
| 192 | ANPDB840 | −8.1 | −7.5 | 239 | ANPDB3003 | −7.3 | −7.3 |
| 193 | ANPDB1651 | −7.4 | −7.5 | 240 | ANPDB402 | −7.3 | −7.3 |
| 194 | ANPDB1918 | −7.4 | −7.5 | 241 | ANPDB5840 | −7.2 | −7.3 |
| 195 | ANPDB1945 | −7.4 | −7.5 | 242 | ANPDB812 | −7.5 | −7.3 |
| 196 | ANPDB3156 | −7.3 | −7.5 | 243 | ANPDB5594 | −7.6 | −7.3 |
| 197 | ANPDB5793 | −7.8 | −7.5 | 244 | ANPDB6433 | −7.8 | −7.3 |
| 198 | ANPDB426 | −7.5 | −7.5 | 245 | ANPDB703 | −8.4 | −7.3 |
| 199 | ANPDB4623 | −7.3 | −7.5 | 246 | ANPDB871 | −8.0 | −7.3 |
| 200 | ANPDB464 | −7.5 | −7.5 | 247 | ANPDB3489 | −7.1 | −7.3 |
| 201 | ANPDB5368 | −7.4 | −7.5 | 248 | ANPDB2294 | −7.2 | −7.3 |
| 202 | ANPDB5369 | −7.2 | −7.5 | 249 | ANPDB2997 | −7.2 | −7.3 |
| 203 | ANPDB5999 | −7.6 | −7.5 | 250 | ANPDB431 | −7.3 | −7.3 |
| 204 | ANPDB2649 | −7.5 | −7.4 | 251 | ANPDB5605 | −7.6 | −7.3 |
| 205 | ANPDB3209 | −7.4 | −7.4 | 252 | ANPDB5685 | −7.1 | −7.3 |
| 206 | ANPDB5588 | −7.1 | −7.4 | 253 | ANPDB5881 | −7.3 | −7.3 |
| 207 | ANPDB1692 | −7.3 | −7.4 | 254 | ANPDB2567 | −7.3 | −7.3 |
| 208 | ANPDB558 | −7.3 | −7.4 | 255 | ANPDB3241 | −7.2 | −7.3 |
| 209 | ANPDB783 | −8.4 | −7.4 | 256 | ANPDB5371 | −7.1 | −7.3 |
| 210 | ANPDB22620 | −7.4 | −7.4 | 257 | ANPDB648 | −8.4 | −7.3 |
| 211 | ANPDB5700 | −7.6 | −7.4 | 258 | ANPDB2969 | −7.2 | −7.3 |
| 212 | ANPDB5160 | −7.4 | −7.4 | 259 | ANPDB5726 | −7.1 | −7.3 |
| 213 | ANPDB5204 | −7.4 | −7.4 | 260 | ANPDB6014 | −7.9 | −7.3 |
| 214 | ANPDB6003 | −7.1 | −7.4 | 261 | ANPDB1289 | −7.2 | −7.2 |
| 215 | ANPDB6047 | −7.4 | −7.4 | 262 | ANPDB1901 | −7.2 | −7.2 |
| 216 | ANPDB1483 | −7.3 | −7.4 | 263 | ANPDB2967 | −7.2 | −7.2 |
| 217 | ANPDB401 | −7.4 | −7.4 | 264 | ANPDB3118 | −7.6 | −7.2 |
| 218 | ANPDB5723 | −7.1 | −7.4 | 265 | ANPDB434 | −7.2 | −7.2 |
| 219 | ANPDB2984 | −7.4 | −7.4 | 266 | ANPDB5288 | −7.2 | −7.2 |
| 220 | ANPDB2992 | −7.3 | −7.4 | 267 | ANPDB5366 | −7.2 | −7.2 |
| 221 | ANPDB5873 | −7.3 | −7.4 | 268 | ANPDB1902 | −7.1 | −7.2 |
| 222 | ANPDB928 | −7.2 | −7.4 | 269 | ANPDB450 | −7.2 | −7.2 |
| 223 | ANPDB386 | −7.4 | −7.4 | 270 | ANPDB4979 | −7.2 | −7.2 |
| 224 | ANPDB404 | −7.4 | −7.4 | 271 | ANPDB5734 | −7.3 | −7.2 |
| 225 | ANPDB379 | −7.3 | −7.3 | 272 | ANPDB6371 | −7.5 | −7.2 |
| 226 | ANPDB6272 | −7.1 | −7.3 | 273 | ANPDB762 | −7.5 | −7.2 |
| 227 | ANPDB761 | −7.5 | −7.3 | 274 | ANPDB874 | −8.1 | −7.2 |
| 228 | ANPDB5580 | −7.4 | −7.3 | 275 | ANPDB1648 | −7.2 | −7.2 |
| 229 | ANPDB5643 | −7.4 | −7.3 | 276 | ANPDB3248 | −7.2 | −7.2 |

**S1 Table.** *Continued*.

| No. | **Compound Code** | **Docking Score (kcal/mol)** | | **No**. | **Compound**  **Code** | **Docking Score (kcal/mol)** | |
| --- | --- | --- | --- | --- | --- | --- | --- |
|  |  | **Standard** | **Expensive** |  |  | **Standard** | **Expensive** |
| 277 | ANPDB4130 | −7.3 | −7.2 | 324 | ANPDB980 | −7.1 | −7.1 |
| 278 | ANPDB4467 | −7.2 | −7.2 | 325 | ANPDB3988 | −7.1 | −7.1 |
| 279 | ANPDB463 | −7.2 | −7.2 | 326 | ANPDB5673 | −7.6 | −7.1 |
| 280 | ANPDB5143 | −7.2 | −7.2 | 327 | ANPDB437 | −7.1 | −7.1 |
| 281 | ANPDB770 | −7.1 | −7.2 | 328 | ANPDB5701 | −7.4 | −7.1 |
| 282 | ANPDB5387 | −7.2 | −7.2 | 329 | ANPDB6181 | −7.7 | −7.1 |
| 283 | ANPDB5893 | −7.1 | −7.2 | 330 | ANPDB2040 | −7.1 | −7.1 |
| 284 | ANPDB6435 | −7.3 | −7.2 | 331 | ANPDB4930 | −7.1 | −7.1 |
| 285 | ANPDB1924 | −7.2 | −7.2 | 332 | ANPDB6474 | −8.1 | −7.1 |
| 286 | ANPDB5465 | −7.9 | −7.2 | 333 | ANPDB1573 | −7.1 | −7.1 |
| 287 | ANPDB6002 | −7.4 | −7.2 | 334 | ANPDB2460 | −7.1 | −7.1 |
| 288 | ANPDB764 | −7.3 | −7.2 | 335 | ANPDB2505 | −7.1 | −7.1 |
| 289 | ANPDB1394 | −7.2 | −7.2 | 336 | ANPDB3366 | −7.8 | −7.1 |
| 290 | ANPDB2447 | −7.1 | −7.2 | 337 | ANPDB4110 | −7.1 | −7.1 |
| 291 | ANPDB348 | −7.1 | −7.2 | 338 | ANPDB6234 | −7.1 | −7.1 |
| 292 | ANPDB4695 | −7.2 | −7.2 | 339 | ANPDB2200 | −7.1 | −7.1 |
| 293 | ANPDB1499 | −7.1 | −7.2 | 340 | ANPDB3049 | −7.3 | −7.1 |
| 294 | ANPDB3285 | −7.1 | −7.2 | 341 | ANPDB5161 | −7.1 | −7.1 |
| 295 | ANPDB3462 | −7.2 | −7.2 | 342 | ANPDB3934 | −7.1 | −7.0 |
| 296 | ANPDB902 | −7.6 | −7.2 | 343 | ANPDB5717 | −7.1 | −7.0 |
| 297 | ANPDB1305 | −7.1 | −7.2 | 344 | ANPDB5934 | −7.1 | −7.0 |
| 298 | ANPDB2974 | −7.2 | −7.2 | 345 | ANPDB6214 | −7.5 | −7.0 |
| 299 | ANPDB313 | −7.1 | −7.2 | 346 | ANPDB2454 | −7.1 | −7.0 |
| 300 | ANPDB6018 | −7.2 | −7.2 | 347 | ANPDB2987 | −7.1 | −7.0 |
| 301 | ANPDB5133 | −7.2 | −7.2 | 348 | ANPDB3069 | −7.1 | −7.0 |
| 302 | ANPDB5467 | −7.2 | −7.2 | 349 | ANPDB4122 | −7.1 | −7.0 |
| 303 | ANPDB5813 | −7.1 | −7.2 | 350 | ANPDB6434 | −7.2 | −7.0 |
| 304 | ANPDB6372 | −7.2 | −7.2 | 351 | ANPDB707 | −8.3 | −7.0 |
| 305 | ANPDB1007 | −7.1 | −7.1 | 352 | ANPDB4621 | −7.1 | −7.0 |
| 306 | ANPDB591 | −7.2 | −7.1 | 353 | ANPDB5146 | −7.1 | −7.0 |
| 307 | ANPDB639 | −7.3 | −7.1 | 354 | ANPDB5379 | −7.1 | −7.0 |
| 308 | ANPDB2154 | −7.1 | −7.1 | 355 | ANPDB5887 | −7.1 | −7.0 |
| 309 | ANPDB4118 | −7.1 | −7.1 | 356 | ANPDB2167 | −7.1 | −7.0 |
| 310 | ANPDB4120 | −7.1 | −7.1 | 357 | ANPDB4043 | −7.1 | −7.0 |
| 311 | ANPDB5064 | −7.1 | −7.1 | 358 | ANPDB429 | −7.1 | −7.0 |
| 312 | ANPDB6228 | −7.5 | −7.1 | 359 | ANPDB2259 | −7.1 | −7.0 |
| 313 | ANPDB6317 | −7.6 | −7.1 | 360 | ANPDB6245 | −7.1 | −7.0 |
| 314 | ANPDB766 | −7.2 | −7.1 | 361 | ANPDB790 | −7.8 | −7.0 |
| 315 | ANPDB2340 | −7.1 | −7.1 | 362 | ANPDB940 | −7.3 | −7.0 |
| 316 | ANPDB5287 | −7.1 | −7.1 | 363 | ANPDB2965 | −7.3 | −6.9 |
| 317 | ANPDB935 | −7.5 | −7.1 | 364 | ANPDB808 | −7.1 | −6.9 |
| 318 | ANPDB4047 | −7.1 | −7.1 | 365 | ANPDB1778 | −7.1 | −6.7 |
| 319 | ANPDB4932 | −7.1 | −7.1 | 366 | ANPDB5593 | −7.9 | −6.7 |
| 320 | ANPDB5801 | −7.5 | −7.1 | 367 | ANPDB6046 | −8.1 | −6.7 |
| 321 | ANPDB5884 | −7.1 | −7.1 | 368 | ANPDB3982 | −7.1 | −6.5 |
| 322 | ANPDB6299 | −7.2 | −7.1 | 369 | ANPDB5103 | −7.1 | −6.3 |
| 323 | ANPDB941 | −7.1 | −7.1 | 370 | ANPDB2859 | −7.1 | −6.1 |

**S1 Table.** *Continued*.

| No. | **Compound Code** | **Docking Score (kcal/mol)** | | **No**. | **Compound**  **Code** | **Docking Score (kcal/mol)** | |
| --- | --- | --- | --- | --- | --- | --- | --- |
|  |  | **Standard** | **Expensive** |  |  | **Standard** | **Expensive** |
| 371 | ANPDB512 | −7.9 | −5.1 |  |  |  |  |

^a^ Data were arranged according to expensive docking scores.
