# Supplementary material for: Exploration of African natural products as VP35 inhibitors to combat Marburg virus infection: Molecular docking, molecular dynamics, and quantum mechanical computations
Source: PLoS One. 2025 Oct 24;20(10):e0334160. doi: 10.1371/journal.pone.0334160 (PMC12551841; doi:10.1371/journal.pone.0334160)
Supplement: S2 Table — (DOCX) [file pone.0334160.s003.docx]

**S2 Table.** Estimated standard and expensive docking scores and MM/GBSA binding energies (in kcal/mol) over 5 ns MD simulations of the promising 68 NPs towards VP35 active site ^a^.

| **No.** | **Compound Code** | **Docking Score (kcal/mol)** | | **MM/BSA Binding Energy (kcal/mol)** |
| --- | --- | --- | --- | --- |
|  |  | **Standard** | **Expensive** |  |
| 1 | ANPDB5109 | –8.7 | −8.3 | –37.1 |
| 2 | ANPDB6426 | –9.0 | −9.1 | −36.5 |
| 3 | ANPDB395 | –8.0 | −8.1 | −35.5 |
| 4 | ANPDB4341 | –8.1 | −8.1 | −32.4 |
| 5 | ANPDB152 | –7.8 | −8.1 | −32.1 |
| 6 | ANPDB2018 | –7.9 | −9.0 | –31.6 |
| 7 | ANPDB383 | –8.4 | −8.5 | –31.0 |
| 8 | ANPDB6357 | –8.0 | −8.3 | –30.5 |
| 9 | ANPDB5459 | –8.9 | −9.0 | –29.9 |
| 10 | ANPDB703 | –8.4 | −8.7 | –29.8 |
| 11 | ANPDB6046 | –8.1 | −8.1 | –29.6 |
| 12 | ANPDB2245 | –8.0 | −8.1 | –29.6 |
| 13 | ANPDB1662 | –7.9 | −8.1 | −29.6 |
| 14 | ANPDB841 | –8.3 | −8.5 | –29.3 |
| 15 | ANPDB851 | –8.1 | −8.1 | −29.3 |
| 16 | ANPDB639 | −12.3 | −12.7 | −29.2 |
| 17 | ANPDB378 | –8.2 | −8.2 | –29.2 |
| 18 | ANPDB840 | –8.1 | −8.2 | –29.2 |
| 19 | ANPDB6425 | –8.4 | −8.4 | –29.1 |
| 20 | ANPDB1660 | –8.4 | −8.4 | –29.0 |
| 21 | ANPDB1783 | –8.6 | −8.7 | –29.0 |
| 22 | ANPDB1837 | –8.0 | −8.2 | –28.3 |
| 23 | ANPDB6433 | –7.8 | −8.9 | –28.1 |
| 24 | ANPDB1919 | –8.2 | −8.2 | –27.7 |
| 25 | ANPDB771 | –8.3 | −8.3 | –27.5 |
| 26 | ANPDB5458 | −8.1 | −9.3 | –27.1 |
| 27 | ANPDB648 | –8.4 | −8.4 | –27.1 |
| 28 | ANPDB1896 | –8.4 | −8.5 | –26.8 |
| 29 | ANPDB6039 | –8.0 | −8.1 | −26.8 |
| 30 | ANPDB6041 | –8.2 | −8.1 | –26.8 |
| 31 | ANPDB6371 | –7.5 | −8.4 | –26.7 |
| 32 | ANPDB6369 | –7.9 | −8.1 | −26.7 |
| 33 | ANPDB6045 | –8.1 | −8.1 | −26.1 |
| 34 | ANPDB2860 | –8.1 | −8.1 | −26.0 |
| 35 | ANPDB3364 | –8.3 | −8.4 | –25.8 |
| 36 | ANPDB6474 | –8.1 | −8.1 | −25.7 |
| 37 | ANPDB465 | –8.7 | −8.7 | –25.2 |
| 38 | ANPDB4740 | –7.9 | −8.1 | −25.0 |
| 39 | ANPDB4294 | –7.5 | −9.0 | –24.9 |
| 40 | ANPDB5386 | –8.0 | −8.1 | −24.7 |
| 41 | ANPDB1922 | –7.9 | −8.1 | −24.4 |
| 42 | ANPDB773 | –8.1 | −8.1 | −24.2 |
| 43 | ANPDB783 | –8.4 | −8.7 | –24.0 |
| 44 | ANPDB5603 | –8.1 | −8.1 | −23.9 |
| 45 | ANPDB5629 | –9.0 | −9.0 | –23.9 |
| 46 | ANPDB6013 | –8.0 | −8.1 | −23.8 |

**S2 Table.** *Continued*.

| **No.** | **Compound Code** | **Docking Score (kcal/mol)** | | **MM/BSA Binding Energy (kcal/mol)** |
| --- | --- | --- | --- | --- |
|  |  | **Standard** | **Expensive** |  |
| 47 | ANPDB871 | –8.0 | −8.1 | −23.8 |
| 48 | ANPDB780 | –8.4 | −8.4 | –23.4 |
| 49 | ANPDB405 | –8.3 | −8.3 | –23.3 |
| 50 | ANPDB707 | –8.3 | −8.3 | –23.2 |
| 51 | ANPDB872 | –8.1 | −8.1 | −23.0 |
| 52 | ANPDB3245 | –7.4 | −8.5 | –22.8 |
| 53 | ANPDB375 | –8.4 | −8.4 | –22.8 |
| 54 | ANPDB6362 | –8.6 | −8.6 | –22.2 |
| 55 | ANPDB6326 | –8.0 | −8.1 | −21.3 |
| 56 | ANPDB643 | –8.3 | −8.3 | –21.2 |
| 57 | ANPDB3468 | −8.0 | −9.2 | −21.1 |
| 58 | ANPDB874 | –8.1 | −8.1 | −20.7 |
| 59 | ANPDB4293 | –8.1 | −8.2 | –20.5 |
| 60 | ANPDB2862 | –8.3 | −8.3 | –20.4 |
| 61 | ANPDB3882 | –8.0 | −8.1 | −19.4 |
| 62 | ANPDB6221 | –8.4 | −8.4 | –19.3 |
| 63 | ANPDB1867 | –8.1 | −8.1 | –19.1 |
| 64 | ANPDB935 | –7.5 | −8.9 | –17.7 |
| 65 | ANPDB2343 | –8.6 | −8.7 | –17.1 |
| 66 | ANPDB3881 | –8.2 | −8.3 | –16.6 |
| 67 | ANPDB5053 | –7.7 | −8.1 | −16.2 |
| 68 | ANPDB1050 | –8.0 | −8.1 | −13.3 |

^a^ Data were arranged according to the MM/GBSA binding energy over 5 ns MDS.
