# Supplementary material for: Exploration of African natural products as VP35 inhibitors to combat Marburg virus infection: Molecular docking, molecular dynamics, and quantum mechanical computations
Source: PLoS One. 2025 Oct 24;20(10):e0334160. doi: 10.1371/journal.pone.0334160 (PMC12551841; doi:10.1371/journal.pone.0334160)
Supplement: S3 Table — (DOCX) [file pone.0334160.s004.docx]

**S3 Table.** Computed docking score, conventional H-bonds, and Δ*G*_binding_ components over 250 ns MDS for ANPDB6426, ANPDB5109, and ANPDB6357 compared to galidesivir and favirpair bound to VP35.

| **Compound Name** | **Docking Score (kcal/mol** | **Conventional H-bonds** | **MM/GBSA binding energy (kcal/mol)** | | | | | | |
| --- | --- | --- | --- | --- | --- | --- | --- | --- | --- |
|  |  |  | **ΔE**_vd_**_W_** | **ΔE_ele_** | **ΔE_GB_** | **ΔE_SUR_** | **ΔG_gas_** | **ΔG_Solv_** | **Δ*G*_binding_** |
| Galidesivir | −6.1 | TYR317  (1.89 Å),  GLN233  (2.66; 1.77 Å). | −13.0 | −4.3 | 29.6 | −28.6 | −17.3 | 1.0 | −16.2 |
| Favirpair | −4.9 | LYS237  (1.72 Å),  VAL234  (2.11 Å) | −12.2 | −4.4 | 27.3 | −25.5 | −16.6 | 2.8 | −13.8 |
| ANPDB6426 | −9.1 | LYS211  (2.14 Å), GLN233  (1.98 Å), LYS237  (2.35 Å) | −50.9 | −13.8 | 33.8 | −7.0 | −64.7 | 26.8 | −37.9 |
| ANPDB5109 | −8.3 | TYR317  (1.70 Å), GLU320  (1.84 Å), LYS287  (1.67 Å) | −37.4 | −31.9 | 40.3 | −5.7 | −69.3 | 34.7 | −34.6 |
| ANPDB6357 | −8.3 | ARG285  (2.25; 2.18 Å) | −42.3 | −12.3 | 26.3 | −5.8 | −54.6 | 20.4 | −34.2 |
